# An Investigation on the Potential of Utilizing Aluminum Alloys in the Production and Storage of Hydrogen Gas

**DOI:** 10.3390/ma17164032

**Published:** 2024-08-14

**Authors:** Reham Reda, Amir Ashraf, Islam Magdy, Mohamed Ragab, Nada Eldabaa, Manar Abo Elmagd, Mohamed Abdelhafiz, Osama El-Banna, Amr Fouad, Hayam A. Aly, Mehdi Tlija, Ahmed T. Soliman, Ahmed Elsayed, Yousef G. Y. Elshaghoul

**Affiliations:** 1 Department of Mechanical Engineering, Faculty of Engineering, Suez University, Suez P.O. Box 43221, Egypt; 2 Central Metallurgical Research and Development Institute (CMRDI), P.O. Box 87, Helwan 11421, Egypt; 3 Department of Industrial Engineering, College of Engineering, King Saud University, P.O. Box 800, Riyadh 11421, Saudi Arabia; 4 Advanced Forming Research Centre, Strathclyde University, Renfrew, Glasgow PA4 9LJ, UK

**Keywords:** aluminum alloys, heat treatment, hydrogen production, electrode surface area, water electrolysis temperature, hydrogen storage and embrittlement

## Abstract

The interest in hydrogen is rapidly expanding because of rising greenhouse gas emissions and the depletion of fossil resources. The current work focuses on employing affordable Al alloys for hydrogen production and storage to identify the most efficient alloy that performs best in each situation. In the first part of this work, hydrogen was generated from water electrolysis. The Al alloys that are being examined as electrodes in a water electrolyzer are 1050-T0, 5052-T0, 6061-T0, 6061-T6, 7075-T0, 7075-T6, and 7075-T7. The flow rate of hydrogen produced, energy consumption, and electrolyzer efficiency were measured at a constant voltage of 9 volts to identify the Al alloy that produces a greater hydrogen flow rate at higher process efficiency. The influence of the electrode surface area and water electrolysis temperature were also studied. The second part of this study examines these Al alloys’ resistance to hydrogen embrittlement for applications involving compressed hydrogen gas storage, whether they are utilized as the primary vessel in Type 1 pressure vessels or as liners in Type 2 or Type 3 pressure vessels. Al alloys underwent electrochemical charging by hydrogen and Charpy impact testing, after which a scanning electron microscope (SEM) was used to investigate the fracture surfaces of both uncharged and H-charged specimens. The structural constituents of the studied alloys were examined using X-ray diffraction analysis and were correlated to the alloys’ performance. Sensitivity analysis revealed that the water electrolysis temperature, electrode surface area, and electrode material type ranked from the highest to lowest in terms of their influence on improving the efficiency of the hydrogen production process. The 6061-T0 Al alloy demonstrated the best performance in both hydrogen production and storage applications at a reasonable material cost.

## 1. Introduction

Future environmental issues and the world’s energy consumption will rise in tandem with population growth and global economic growth. Coal, oil, and natural gas are the primary fossil fuel resources that provide most of the world’s energy. However, the depletion of fossil fuels and the negative environmental effects of using fossil fuels have forced extensive research into cutting-edge technologies for the utilization and storage of clean, sustainable energy sources like wind and solar power [1,2,3,4,5]. Even though using solar and wind energy systems—or systems like them—is appealing, neither system is appropriate for use on its own because of variations in day and night and seasonal variables that primarily affect its efficiency [1].

Hydrogen is considered one of the “fuels of the future” for structures, portable devices, automobiles, rocket fuel for space missions, etc. Hydrogen is an effective and environmentally friendly energy carrier for renewable resources with a high energy density of 33.3 kW h/kg [3,4,6,7,8,9]. Hydrogen can be utilized in internal combustion engines to generate work, like moving different transportation systems. However, the utility can be greatly increased if this is accomplished by using fuel cells to convert hydrogen energy into electrical energy, which can then be utilized to drive an electrical motor [3,5,6,7,10]. Hydrogen has the potential to serve as an energy storage medium [3,7,11,12]. For instance, excess electrical energy might be electrolyzed and stored as hydrogen during off-peak hours, preventing waste [3,11,12]. As a result of recent technological advancements, hydrogen energy is becoming a more viable energy source. Soon, hydrogen will take the place of electricity as the main energy source in the sustainable energy future [7,10,12]. Addressing hydrogen generation and storage is essential for the widespread adoption of hydrogen [10]. There are many methods of hydrogen generation routes, including biological, chemical, electrochemical (water electrolysis, photoelectrochemical, halide electrolysis, H_2_S electrolysis), and thermal methods [4,10,11,12]. The cost of producing hydrogen and its associated emissions can vary greatly depending on the type of energy utilized in production.

Different colors are used to distinguish the different approaches for producing hydrogen based on the principal energy sources [11,12]. The hydrogen produced by coal gasification or steam reforming of natural gas without carbon capture is represented by the grey hydrogen [11,12]. The main drawback of grey hydrogen is that it produces a lot of CO_2_ during the hydrogen generation process. Blue hydrogen is generated by reforming the methane steam with carbon captured. Grey hydrogen’s emissions are barely halved by blue hydrogen [12]. Blue hydrogen is seen as a technique that bridges the gap before a complete switch to green hydrogen [11,12]. Methane pyrolysis yields turquoise hydrogen with solid carbon in the form of carbon nanotubes or filamentous carbon, which can be stored more easily or used in subsequent processes, hence leaving less of a carbon footprint [11,12]. Green hydrogen is generated by electrolyzing water using electricity derived from renewable energy sources. Particular attention is paid to this type of hydrogen for moving towards a more environmentally friendly energy and transportation infrastructure [3,11,12]. Orange hydrogen is produced through electrolysis using grid electricity [11,12]. The sources of energy utilized in hydrogen generation have the most effect on the amount of CO_2_ emitted. There are essentially no corresponding CO_2_ emissions for the routes if water is the feedstock (e.g., green and orange hydrogen) [11]. Alkaline and polymer electrolyte membrane electrolyzers are mainly employed for green and orange hydrogen generation [11]. The characteristics of orange hydrogen are mostly determined by the energy mix of the entire country that powers the electrolyzer as well as the cost of that electricity [11].

Water electrolysis has been seen as an appealing strategy in the context of clean energy sources [3,4,6,13,14]. Water cannot be directly converted into hydrogen and oxygen due to its high electrical resistance. Water electrolysis is a process that produces solely hydrogen and oxygen when an electrical current is passed through water-based solutions; as a result, no greenhouse gas emissions are produced if the electrolysis cell’s power is generated from renewable resources [3,6,14]. These processes include the anodic electro-oxidation reaction, also known as the Oxygen Evaluation Reaction (OER), and the cathodic electro-reduction reaction, also known as the Hydrogen Evaluation Reaction (HER) [1,14]. In terms of mass-charge transfer and electrode dynamics, these reactions are extremely slow in practice. The development of affordable materials to boost the system’s kinetics and reduce the consumed electrical energy are crucial stages towards rendering the system economically viable [1,4,10,14,15]. The electrolyzer’s overall efficiency is often less than 40%. Therefore, the cost of producing hydrogen must be as low as feasible for the future hydrogen economy. Many efforts were made to enhance the efficiency and reduce the consumed energy of the water electrolyzers, including the development of alternative materials for electrodes [1,13,14,15] and decreasing the electrode gap [10].

The influence of the electrode materials in the electrolyzer on cost and efficiency was an issue of limited previous studies [1,3,6,10,13]. In comparison to carbon steel, Okonkwo et al. [13] found that stainless steel 316L electrode has better hydrogen production and efficiency. Symes et al. [3] found that cost-effective electrochemical behavior is exhibited by nickel and stainless-steel electrodes. Additionally, they stated that compared to unpolished electrodes, polished electrodes show an increased rate of gas production. Because oxide layers reduce the number of active reaction sites available for bubble nucleation, they increase resistance in the electrolyzer [3]. Due to their susceptibility to oxidation, Al alloys can be used to create a high-performing auxiliary hydrogen production system in the electrolyzer when used as an anode [1].

Park et al. [15] examined how the cathode area and shape affect the critical current density and breakdown voltage in water electrolysis. They observed that regardless of the cathode’s shape, the breakdown voltage increased as the cathode area increased, and the cathode shape significantly affected the critical current density.

Increasing the temperature promotes the reaction kinetics in the water electrolyzer as the heating alters the physicochemical properties of water. The dielectric constant (polarity), surface tension, and viscosity of water decrease while the concentration of OH^−^ and H^+^ ions increases when water is heated. As temperature and pressure rise, the voltage needed for an electron to cross the Helmholtz energy barrier decreases, which lowers the potential needed to produce hydrogen gas. The generation of hydrogen gas under high pressure and temperature has been previously studied. However, because of the issues with high temperature resistance referencing electrodes and gas separation, not enough development has been made. Reactor designs that are both affordable and long-lasting are also crucial in these kinds of systems [1].

The beneficial impacts of increasing temperature and decreasing the distance (between electrodes) on energy savings were reported by Stojic et al. [10]. The influence of electrode spacing on hydrogen production was examined by Okonkwo et al. [6]. They found that the rate of the electrochemical reaction, the production of hydrogen, and efficiency are all increased when the distance between the immersed electrodes is reduced.

The effect of KOH electrolyte concentration on the HHO generator’s performance at various stainless steel 316L electrode surface textures was investigated by Ridhwan et al. [2]. They found that increasing the KOH concentration in conjunction with various electrode surface textures improves the HHO generator’s performance. Varying the surface texture results in increasing the electrode surface area, hence improving the HHO generator’s performance.

Despite an abundance of research and recommendations surrounding water electrolysis, correlating the electrode material behavior, efficiency, and cost is very limited in the literature. The requirements of selecting the electrode material necessitate careful consideration. Moreover, examining the influence of single electrode surface area is rarely covered.

The success of the hydrogen energy markets depends on the efficient storage of hydrogen. Compressed gases, cryo-compressed gases, refrigerated liquefied gases, and solid hydrides are the four different ways of hydrogen storage [7,16]. Four different kinds of pressure vessels can be used to store hydrogen. Type I pressure vessels (PVs) are made of metal. Type II PVs are made up of a cylindrical metallic portion covered in a fiber-resin composite and a thick metallic liner hoop, while in Type III PVs the metallic liner is fully overwrapped with fiber-resin composite. Type IV PVs employ a polymer liner fully wound with composite [7,8,16]. Because composite pressure vessels are more expensive than metallic ones, their market share is still quite narrow. The desired application specifies the storage method, necessitating a trade-off between cost competitiveness and technical performance.

The design of all pressure vessels must consider the operating conditions, lifetime, external loads (such as mechanical impacts), etc. Failure modes must also be considered while selecting materials [16]. Type I PVs as well as the metallic components of the other PVs, such as the liner and boss, are frequently made of Al alloys or various forms of high Cr-Ni-Mo alloyed steels [8,16,17]. In contrast to the body-centered cubic (BCC) crystal structure, hydrogen diffusion in Al lattice is negligible because of its stable face-centered cubic (FCC) crystal structure at ambient temperature [17].

Many ways are utilized to construct type I pressure vessels as well as type II and III liners from tubes or plates that are deep-drawn to take on the required shape. Then, hot spinning is used to produce the neck and ports. Heat treatments are then applied to provide the necessary mechanical characteristics [16]. For industrial applications in metallic type I PVs, hydrogen is maintained at 20–30 MPa [16]. Compressed hydrogen gas is utilized in stationary applications because it takes up a large storage space. Hydrogen storage has numerous stationary uses, including backup power generation, off-grid power supplies, and household power generators [16].

Under high pressure, hydrogen tends to adsorb and dissolve at material surfaces; the atomic hydrogen then diffuses into the material and interacts with lattice defects, causing embrittlement, i.e., reduction in ductility and/or toughness [7,8,18,19]. In general, hydrogen embrittlement (HE) affects metallic materials that are used in the manufacturing of storage tanks when they come into contact with hydrogen under an applied pressure [8]. This can lead to a premature crack and deterioration of mechanical properties. Stress corrosion cracking, or H-induced cracking, is the cause of it. Both the industry and academia have made significant efforts to address this issue by expanding the understanding of the HE mechanisms, manufacturing processes for alloys, component assembly, and suitable mechanical testing [7,16]. The safety of hydrogen storage systems depends on the proper selection of materials that are appropriate for each component. This has to do with fittings, valves, filling connectors, walls of storage containers, pipelines, etc. [7].

If hydrogen does not diffuse far enough into a specimen, HE does not happen [20]. The chemical composition and heat treatment have been well recognized as a popular method for modifying the mechanical characteristics and hydrogen embrittlement behavior of aluminum alloys due to their evident impact on the kind and size of precipitates [5,8]. The amount and kind of precipitates are believed to be highly associated with embrittlement susceptibility. It is essential to investigate the structural and chemical composition of the hydrogen storage material to comprehend the hydrogen embrittlement mechanism of the storage tank [7]. The majority of the previous research dealt with the impact of chemical compositions and microstructures on hydrogen embrittlement, focusing on austenitic stainless steels, and a limited number of studies have been conducted for aluminum alloys.

Moshtaghi et al. [5] found that the yield and ultimate tensile strength of an Al–Zn–Mg–Cu alloy were improved with the increase in solution treatment temperature and that the variations in the test environment had little effect on yield and ultimate tensile strengths, whereas the overall elongation significantly decreased during tensile testing in the humid air as compared with in dry nitrogen gas. They stated also that the solute segregation to the grain boundary affects the specimens’ susceptibility to hydrogen embrittlement. Compared to high and low solution treatment temperature conditions, the specimen with a moderate solution treatment temperature (420–450 °C) is more resistant to hydrogen embrittlement [5].

The effect of hydrogen embrittlement on the impact toughness of Al-Mg MIG weld was investigated by Han et al. [19]. They observed that the decohesion of contaminants from the matrix and the creation of porosity resulted in a drop in impact toughness as the hydrogen concentration increased. They stated that impact toughness is highly susceptible to alterations in microstructure and macroscopic flaws. The presence of impurities in aluminum alloys could make them more brittle, which would lower their impact toughness.

Through in situ hydrogen charging at various strain rates, the influence of hydrogen on the mechanical characteristics of AA 7075-T6 was investigated by Dey and Chattoraj [20]. Despite the cathodic charge, the alloy formed a surface hydroxide. The alloy’s hydrogen embrittlement is inhibited by this oxide layer. While hydrogen embrittlement was considerable at the slowest strain rates, the film interfered and reduced hydrogen entrance at intermediate strain rates.

As mentioned above, thanks to their high strength-to-weight ratio and comparatively low cost, Al alloys can be employed as electrodes for hydrogen generation by water electrolysis and in hydrogen storage tanks, either as a main structure of PV in type 1 or as a liner in type 2 and type 3 PVs. In the current study, various commercial Al alloys were compared and evaluated for their ability to generate hydrogen when employed as electrodes in an electrolyzer. The effect of the electrode surface area and electrolysis temperature on the hydrogen flow rate, energy consumption, and electrolyzer efficiency were studied. Additionally, these alloys are tested for hydrogen embrittlement for compressed hydrogen gas storage applications through a Charpy impact toughness test before and after H charging. Moreover, the fracture modes for the different studied Al alloys were investigated using SEM. Finally, the best alloy for both electrode and storage applications was determined, and a correlation was made to their structural constituents.

## 2. Materials and Method

### 2.1. Materials and Specimens Preparation

In the current work, four distinct Al alloys with dimensions of 90 × 50 × 3 mm^3^ were used, namely, 1050, 5052, 6061, and 7075 Al alloys. The alloys were supplied by Helwan Company for Non-Ferrous Industries and made using a direct strip casting (DSC) process. The chemical compositions of the Al alloys under investigation were examined using optical emission spectroscopy (OES) and presented in Table 1. Table 2 displays these alloys’ costs according to suppliers’ data for the current year. Low surface area electrodes measuring 50 × 40 mm^2^ and high surface area electrodes measuring 90 × 50 mm^2^ were cut from each alloy using an electrical discharge machine (EDM). Moreover, the specimens for Charpy impact toughness tests were also cut from each alloy according to ASTM E23 [21].

### 2.2. Heat Treatment

**Figure 1:**
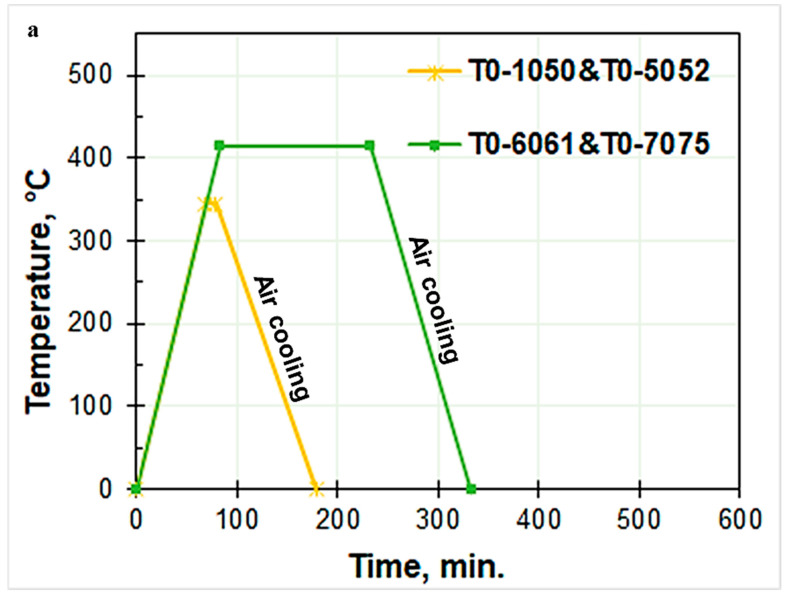

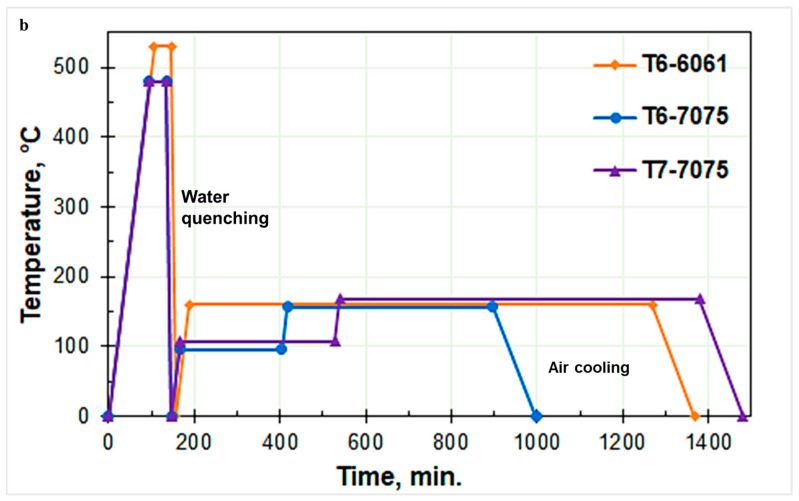
Schematic diagrams of the heat treatment cycles performed on the studied Al alloys: (**a**) T0 annealing treatment for all studied alloys and (**b**) T6 and T7 heat treatments for 6061 and 7075 Al-alloys.

As shown in Figure 1, the Al alloys experienced various typical heat treatment regimes. With a constant heating rate of 5 °C/min, an induction furnace was used to carry out the heat treatment. The T0 annealing treatment, which involves heating the alloys to 345 °C and holding them for 10 min before air cooling, was applied to both the 1050 and 5052 non-heat-treatable aluminum alloys (Figure 1a). T0 and T6 heat treatments were applied to the 6061 Al alloy. T0, T6, and T7 heat treatments were applied to the 7075 Al alloy. T0 for the 6061 and 7075 Al alloys involved heating to 415 °C, holding for 150 min, followed by air cooling (Figure 1a).

T6 for 6061 Al alloy involved heating to 530 °C and holding for 40 min, followed by water quenching; after that, the alloy was heated to 160 °C and held for 40 min, followed by air cooling. T6 for the 7075 Al alloy, on the other hand, required heating to 480 °C and keeping it for 40 min, then quenching in water, with subsequent heating to 96 °C and holding for 240 min, followed by heating to 157 °C and holding for 8 h, and finally cooling in air (Figure 1b). Only the 7075 Al alloy underwent the T7 heat treatment, which included heating to 480 °C and holding for 40 min, followed by water quenching, then heating to 107 °C and holding for 360 min, then heating to 168 °C and holding for 840 min, and finally air cooling (Figure 1b).

### 2.3. Structural Analysis

The heat-treated specimens were ground and polished for X-ray diffraction (XRD) analysis. The constituents of each alloy were identified with Cu kα radiation in the 0–100° range at a scanning rate of 0.2°/min.

### 2.4. Water Electrolysis and Measurements

**Figure 2:**
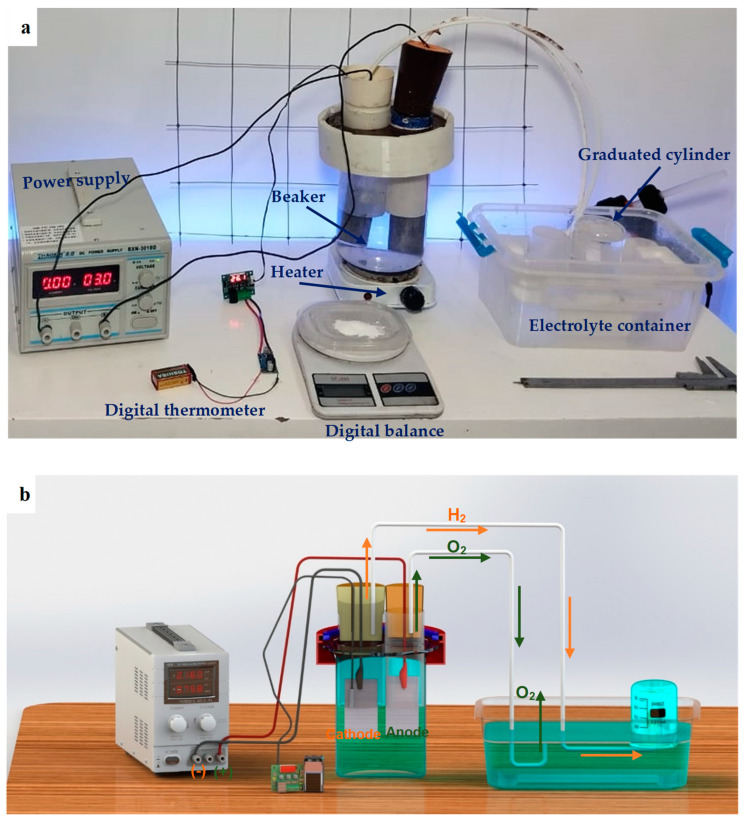
A real photo of the experimental setup with identified components (**a**) and a schematic representation of the hydrogen gas generation from the water electrolysis process (**b**).

The electric circuit and experimental setup for the two-electrode electrolyzer utilized in this work are shown in Figure 2. Following heat treatment, emery paper and alcohol were used for mechanically polishing the electrode surfaces. The influence of the electrode’s surface area was also examined in addition to the electrode material. Table 3 displays the several sizes of the examined anode and cathode electrodes. Four cases were examined, including employing a low surface area for both electrodes, a high surface area for both electrodes, a high surface area for an anode, and a high surface area for a cathode.

A 200 mL beaker was used as the body of the electrolyzer. A 60 mm electrode spacing was employed to set up the electrolyzer to minimize the ohmic resistance in the electrolyzer and to enable the separation of the produced hydrogen and oxygen gases. Throughout the test, the electrodes were placed in 2 L of 35 g/L sodium chloride (NaCl) solution. This solution was used to avoid the side effects of unsafe acids and alkalines. In every experiment, the anode and cathode were two electrodes made of the same material. The power supply is connected to the electrolyzer to provide the electricity with a constant input voltage of 9 volts for a fixed duration of 10 min. The oxygen is released into the atmosphere, whereas the hydrogen is collected in a container. A water-filled container was continuously filled with cathodically evolved hydrogen gas. To compute the volumetric flow rate of the generated hydrogen, the volume change measurement of water in a 50 mL graduated cylinder was used to measure the produced hydrogen gas volume at a constant time of 5 min, as demonstrated in Figure 2. The overall current and voltage through the cell were observed. The energy consumption and the electrolyzer efficiency were measured for a given temperature. The experiments on hydrogen evolution were conducted at room temperature and 60 °C. The electrolyte temperature is raised using a heater, and the temperature is monitored with a digital thermometer. This study deals with orange hydrogen as the electrical grid serves as the energy source for the water electrolysis process.

### 2.5. Hydrogen Charging and Hydrogen Embrittlement Evaluation

Various Al alloys were examined, and their hydrogen embrittlement (HE) was assessed. By cathodic charging, atomic hydrogen is purposefully introduced into metallic materials to investigate the effects of hydrogen embrittlement. Each charging experiment was conducted at 60 °C for 20 min. Three impact specimens from each alloy condition were employed as a cathode with an anode of as-received 1050 Al alloy of 90 × 50 × 3 mm^3^. The susceptibility to HE was assessed using the Charpy impact test.

To avoid unintentional hydrogen desorption, the sample was immediately stored in a vessel filled with liquid nitrogen following the hydrogen charge and held there until the impact test began. Less than 30 min passed between removing the sample from the vessel and doing the impact test.

Charpy impact tests were performed for all specimens, i.e., uncharged and H-charged specimens. All specimens’ fracture morphology was investigated using a scanning electron microscope (SEM) to identify the fracture characteristics.

## 3. Results and Discussion

### 3.1. Structural Investigation

**Figure 3:**
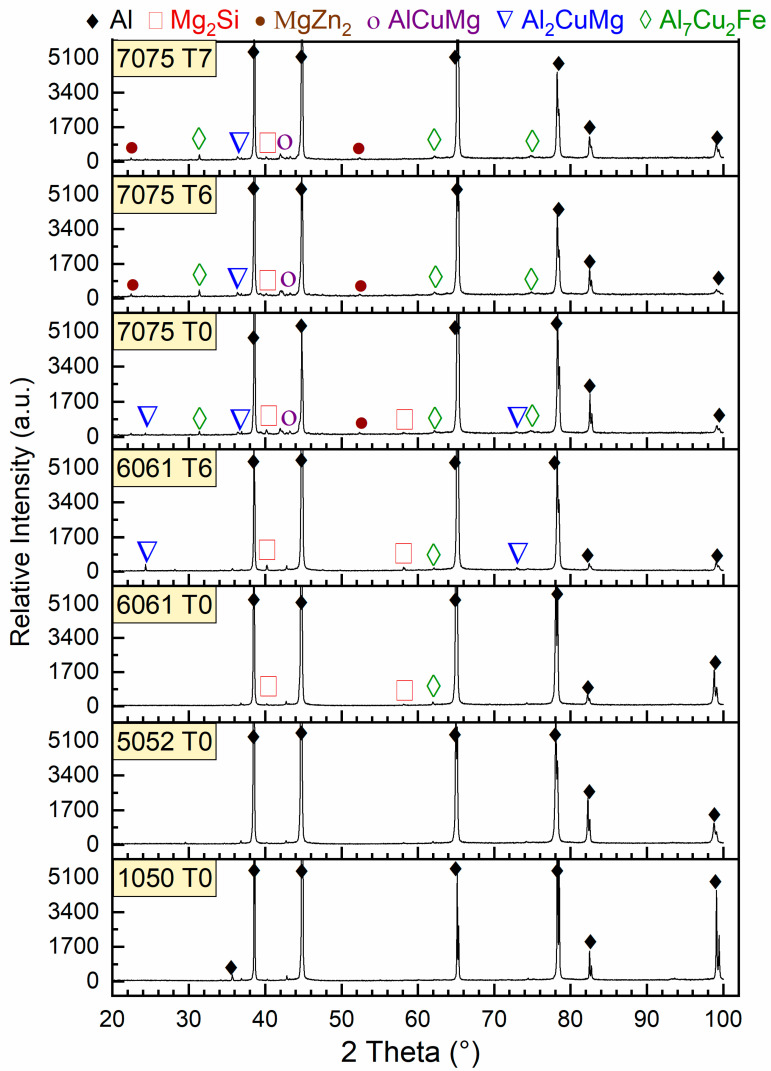
XRD profiles of the studied Al alloys.

Figure 3 shows the XRD peak profiles for the studied alloys. Only pure α-Al peaks are detected in both 1050-T0 and 5052-T0 Al alloys, according to the XRD data. Since these alloys are non-heat-treatable alloys, they do not have any precipitates. The Mg_2_Si and Al_7_CuFe, and α-Al peaks phases are revealed in the XRD data for both the 6061-T0 and 6061-T6 Al alloys, in addition to Al_2_CuMg in the 6061-T6 Al alloy. Several intermetallic compounds, such as Al_2_CuMg, Al_7_Cu_2_Fe, AlCuMg, Mg_2_Si, and MgZn_2_, are detected by XRD analysis of the 7075-T0, 7075-T6, and 7075-T7 Al alloy. These compounds are varied in quantity and size depending on the processing conditions, as stated previously [22,23,24,25].

### 3.2. Al Alloy Electrodes for Hydrogen Gas Generation Applications

When producing hydrogen, employing aluminum electrodes has a lot of benefits. Aluminum is a cheap, recyclable substance that is abundant in the natural world. Moreover, the aluminum anode inhibits oxygen gas from forming in the electrolyzer, promoting the creation of cleaner hydrogen. Therefore, water electrolysis will be less expensive and eliminate the need for gas separation procedures [1,6]. An electrical current is introduced between the electrodes during water electrolysis, splitting the molecules into hydrogen and oxygen gases. The water electrolysis reactions employing aluminum electrodes in the slightly alkaline and alkaline solutions are as follows [1,2,6,13]:

Cathode: 4H_2_O + 4e^−^ →2H_2_ + 4OH^−^

Anode: 4OH^−^ →O_2_ + 2H_2_O + 4e^−^

Al (s) + OH^−^ + H_2_O → AlO_2_^−^ + 3/2H_2_ (g)

Because the material of the electrodes can affect hydrogen production through the interaction between the electrodes and the electrolyte, the electrode material is an important consideration for hydrogen generation through water electrolysis. Reducing energy loss and equipment costs is necessary for water electrolysis to become a more efficient and competitive process; therefore, the practical cell voltage should be lowered as much as possible to increase the cost-benefit of electric energy [14]. In light of this, various electrode materials were examined in this work to determine which material produces a high hydrogen flow rate at a low amount of energy consumption. The influence of electrode surface area and electrolysis temperature were also studied.

#### 3.2.1. Effect of Electrode Material and Surface Area

**Figure 4:**
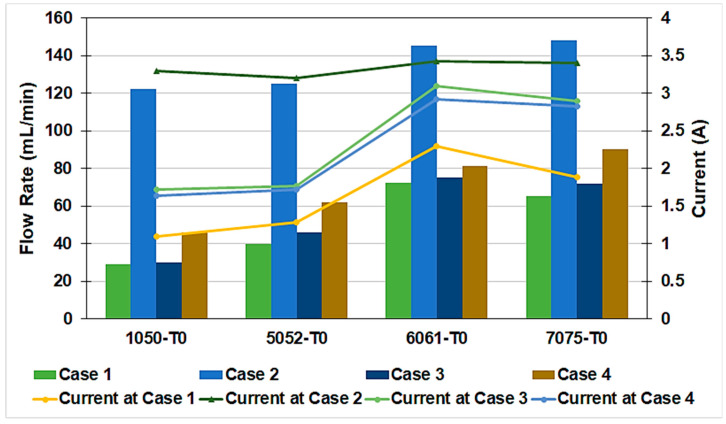
Effect of the electrode materials and surface area on the hydrogen flow rate and the current at a constant voltage of 9 V.

In the current study, the effect of electrode material and surface area on the hydrogen flow rate and the current at a constant voltage of 9 V were studied, as shown in Figure 4. Regarding the electrode material, the four Al alloys 1050, 5052, 6061, and 7075 in the annealed temper condition T0 were tested as electrodes in a water electrolyzer at different surface areas. Table 3 presents the different studied cases of the electrode surface area. In case 1, two low surface area electrodes of 50 × 40 mm^2^ were used, while in case 2, two high surface area electrodes of 90 × 50 mm^2^ were tested. In case 3, a low cathode surface area of 50 × 40 mm^2^ with a high anode surface area of 90 × 50 mm^2^ was examined, while in case 4, a high cathode surface area of 90 × 50 mm^2^ with a low anode surface area of 50 × 40 mm^2^ was examined. The aim of these procedures was to study the influence of each electrode surface area on the performance of the water electrolysis process.

In the annealed temper conditions, 7075 and 6061 Al alloys result in the highest hydrogen flow rate at room temperature and a constant voltage of 9 V, as shown in Figure 4. The 1050 Al alloy shows the lowest hydrogen flow rate while the 5052 Al alloy demonstrates an intermediate performance. It was observed that the utilization of the low electrode surface area of both anode and cathode (case 1) has a similar performance to case 3, utilizing high anode and low cathode surface areas. Case 2, employing a high electrode surface area, reveals the highest hydrogen flow rate, followed by case 4 utilizing low anode and high cathode surface areas. Therefore, increasing the surface area of the cathode has a beneficial influence on the generated hydrogen flow rate.

Moreover, the current results reveal that for both 1050 T0 and 5052 T0 Al alloys, the current is the lowest in case 1 and highest in case 2 compared to other studied cases. This is attributed to increasing the electrode surface area at a constant voltage and temperature. Similar results were obtained previously [3]. It can also be observed that the current in case 4 is lower than in case 3, although a higher hydrogen flow rate is generated in case 4. This means that increasing the cathode surface area over the anode surface area results in a higher hydrogen flow rate at lower energy consumption.

The electrolyzer efficiencies for the studied electrode materials and surface areas at room temperature are presented in Table 4. Increasing efficiency means increasing the amount of hydrogen produced at lower energy consumption. At room temperature and in the annealed temper conditions, 7075 and 6061 Al alloys reveal the highest efficiencies, followed by the 5052 Al alloy. The 1050 reveals inferior performance. Moreover, increasing the surface area of the cathode more than the anode has the most beneficial influence on the process efficiency, as shown in Table 4.

#### 3.2.2. Effect of Electrode Material and Electrolysis Temperature

**Figure 5:**
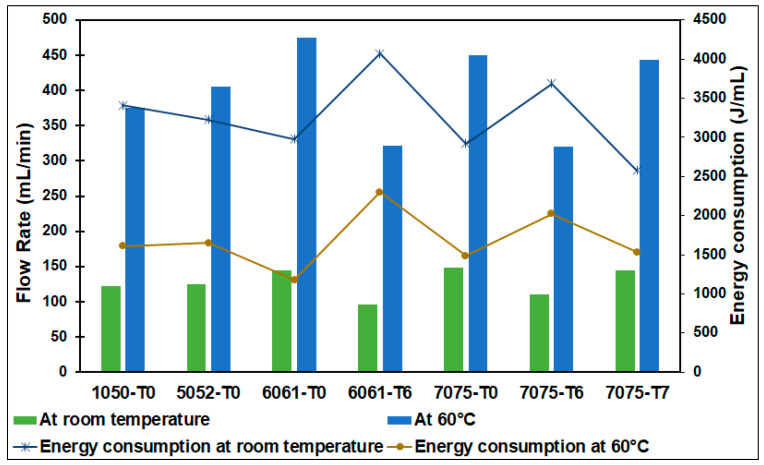
Effect of the electrode material and electrolysis temperature on the hydrogen flow rate and energy consumed.

The effect of the electrode material and electrolysis temperature on the hydrogen flow rate and energy consumed per 1 mL of hydrogen gas evolved is shown in Figure 5. The experiments were carried out at both room temperature and at 60 °C. In these experiments, higher anode and cathode surface areas were applied (case 2) for different temper conditions, namely, 1050-T0, 5052-T0, 6061-T0, 6061-T6, 7075-T0, 7075-T6, and 7075-T7 Al alloys.

The following formula is used to determine the energy consumption (Q) in the hydrogen generation process during water electrolysis [1,10,14]:

Q = I × U × t (1)

where U represents the electrolyzer’s potential (volt), I is the overall current (amperes), and t denotes the time of evolution of a certain volume of hydrogen gas (seconds).

By rising temperature, the consumed energy significantly decreases below the consumed energy at room temperature, as shown in Figure 5. This is attributed to the fact that a higher temperature results in a faster reaction rate, which raises the current for a given voltage or decreases the voltage required for a specific current, as stated by Stojic et al. [10]. In the current work, the current increases and the voltage decreases as compared to their values at room temperature. With increasing temperature, ionic conductivity and the electrolyte’s surface reactivity both rise; moreover, the energy loss via electrode polarization is reduced. It was shown that the magnitude of the current, voltage, and surface response between the electrodes and the electrolyte supports the breaking up of water molecules during electrolysis [13].

Utilizing the 6061 T0 Al alloy as the electrode material at a high temperature of 60 °C produces the highest hydrogen flow rate of 475 mL/min. At a low energy consumption of 1182 J/mL, followed by 7075 T0 and 7075 T7, Al alloys produce 450 and 443 mL/min hydrogen flow rates at 1488 J/mL and 1536 J/mL energy consumption, respectively. Intermediate performance is revealed by 5052 T0 and 1050 T0, while the lowest performance is presented by 6061 T6 and 7075 T6 Al alloys.

#### 3.2.3. Efficiency of the Electrolyzer

The electrolyte concentration, the applied potential, and the electrode surface area are some of the elements that affect the electrolyzer’s efficiency [3]. To assess the electrolyzer’s efficiency in hydrogen production (η), the ratio of the energy produced by the hydrogen gas to the amount of electrical power consumed to generate it is estimated using the following equation [4]:

(2) $$\eta =\frac{V_{H2}\times \rho_{H2}\times {LHV}_{H2}}{V\times I\times t}$$

V_H2_ is the volume of hydrogen (m^3^), LHV_H2_ is a low heat value of hydrogen equal to 119.93 × 10^6^ J/kg, and ρ_H2_ is the density of hydrogen gas equal to 0.0838 kg/m^3^. V is the voltage (volts), I is the current (amperes), and t is the time (seconds), which were measured experimentally.

**Figure 6:**
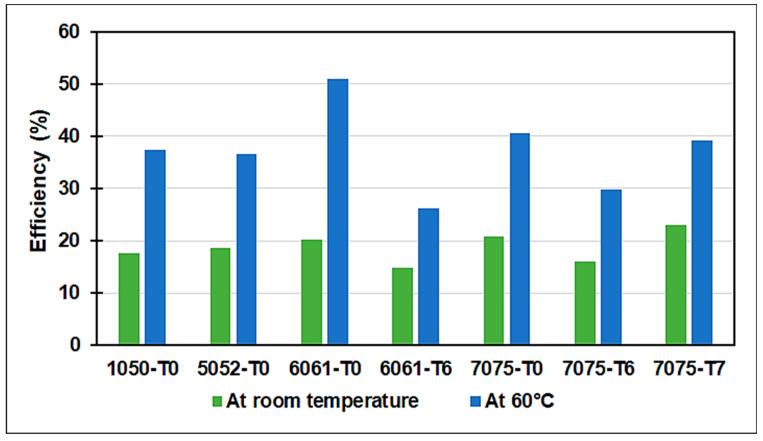
Effect of the electrode material and electrolysis temperature on the efficiency of the electrolyzer during hydrogen gas production.

Figure 6 demonstrates the effect of the electrode material and electrolysis temperature on the efficiency of the electrolyzer during hydrogen gas production. As presented, raising the temperature of the water electrolysis has a significant influence on the electrolyzer efficiency. This is explained by the fact that a higher temperature accelerates the reaction rate, which raises the ionic conductivity and reactivity of the electrolyte; hence, it results in a higher hydrogen flow rate at lower energy consumption, as described in the above section. Relating the efficiency with the cost of materials (Table 2) and the cost of heat treatment, it is found that utilizing 6061 T0 Al alloy at a relatively high temperature of water electrolysis reveals high efficiency at a moderate cost of the material and processing. Followed by 1050 T0, 5052 T0, 7075 T0, and 7075 T7 Al alloys as they reveal similar efficiency at low and high temperatures. According to our findings, the 6061 T0 Al alloy electrode produces the highest hydrogen flow rate at low energy consumption and high efficiency, which makes it a superior material for water-splitting electrolysis. From a cost point of view, 1050 T0 and 5052 T0 Al alloys have additional advantages. The 6061 T6 and 7075 T6 Al alloys reveal weak performance at high material and processing costs; therefore, they should be excluded from being used as electrodes in water electrolysis for hydrogen generation. Generally, it was observed that the temper condition (T0) is the best-recommended treatment for the electrode material.

#### 3.2.4. Performance of Al Alloy Electrodes for Hydrogen Gas Generation

Clean hydrogen has the potential to replace traditional fossil fuels and contribute to carbon-free energy. Water electrolysis is gaining a lot of attention as a method for producing hydrogen without greenhouse gas emissions. However, because of the overpotential, water electrolysis needs a high voltage to provide a suitable current. The high cost of electricity required prevents water electrolysis from being used commercially. Rising the temperature increases hydrogen generation, reduces the energy consumption, and promotes the process efficiency. Because heat is a less expensive energy source than electricity and because the electrolysis reaction is more effective at higher temperatures, high-temperature electrolysis is more economically efficient than conventional room-temperature electrolysis. Nonetheless, the materials used for the electrodes had an impact on the quantity of hydrogen generated. Utilizing an aluminum anode encourages the production of clean hydrogen as it impedes the formation of oxygen gas in the electrolyzer [1,10,14].

To identify the most influencing factors on the efficiency of hydrogen gas generation, a sensitivity analysis was performed. The one-factor-at-a-time (OAT) approach is a widely used and straightforward method for monitoring variations in the result of sensitivity analysis. It involves altering a certain variable one at a time while maintaining the other values constants. This improves the results’ comparability. This seems like a logical procedure since any changes detected in the output will be caused by the single changed parameters. Moreover, it is possible to determine which variable had the strongest influence [26]. 

**Figure 7:**
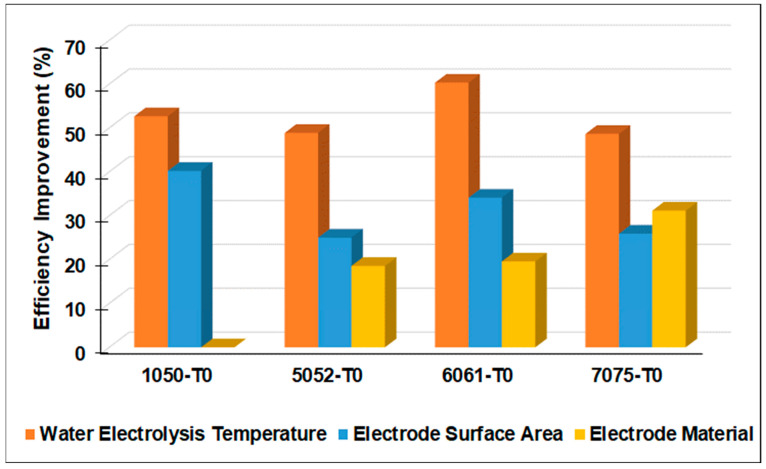
OAT sensitivity analysis of the influence of the water electrolysis temperature, the electrode surface area, and the electrode material on the improvement of hydrogen gas generation efficiency.

By plotting the individual influence of the electrolysis temperature, electrode surface area, and electrode material on the process efficiency improvement, the most influencing factor on the process efficiency can be determined. Figure 7 presents the OAT sensitivity analysis of the influence of the water electrolysis temperature, the electrode surface area, and the electrode material in the annealed temper condition (T0) on the hydrogen gas generation efficiency for each studied Al alloy. It is obvious that raising the water electrolysis temperature is the most influencing factor on the process efficiency, followed by the electrode surface area. Contrary to predictions, the electrode material’s effect had minimal influence as compared to the other studied factors. These results were observed for 1050, 5052, and 6061 Al alloys. The 7075 Al alloy reveals different behavior as the electrode material type has a stronger effect than the electrode surface area.

### 3.3. Hydrogen Embrittlement of Al Alloys for Hydrogen Gas Storage Applications

One major obstacle to the widespread development and commercialization of hydrogen technology to establish a green society is the material deterioration of hydrogen storage tanks by hydrogen embrittlement [7]. Al alloys are employed as the primary vessel in Type 1 pressure vessels or as liners in Type 2 or Type 3 pressure vessels. Several attempts have been made to enhance hydrogen storage facilities and lessen hydrogen embrittlement. Surface coating and material microstructural changes have been at the forefront of this strategy. The current work adapted the second technique, which involves tailoring the alloy microstructure via chemical composition and heat treatment. The performance of hydrogen pressure vessels can be improved by choosing the proper materials and tailoring the structural characteristics by diminishing the embrittlement of hydrogen [7]. It is worth mentioning that the type of hydrogen storage tank and its storage technique can have an immense effect on the hydrogen fuel cost [7]. For the shipping, storage, and fuel cell cars of hydrogen, high-pressure hydrogen tanks are essential. However, because of problems with embrittlement in storage materials, low hydrogen density poses both technical and financial difficulties. Deep knowledge necessitates surface and structural alterations of the storage material [7].

When hydrogen atoms are absorbed in materials, they segregate at defect sites, and high gas-pressure hydrogen molecules are then formed. At sites of metallurgical instability, this pressure increases, causing hydrogen-induced cracking and catastrophic failures at stresses lower than the yield stress of the material. This process is known as hydrogen embrittlement [7].

In high-strength alloys, hydrogen embrittlement is a phenomenon that can lead to crack propagation, mechanical property loss, and catastrophic failure. It happens when hydrogen, storage materials, and high-pressure gaseous environments interact electrochemically [7,17]. Several techniques have been used to illustrate the hydrogen embrittlement in materials. Among these, hydrogen changed the micro-fracture mode, concentrating on the material’s fracture transition mode from ductile to brittle [7,17,19,20].

A process for introducing H to aluminum alloys for examination is called H charging. In this process, the specimens are placed as a cathode in the water electrolysis cell. As the hydrogen is evolved at the cathode during water electrolysis, resulting in exposing the specimen to hydrogen for a predetermined period of time. The H charging process becomes more efficient at a high temperature, as rising temperatures enhance the chemical reaction, hydrogen flow rate, and H diffusion to the specimen. Hydrogen can be localized at either trap sites or lattice sites once it has diffused into the lattice [17].

**Figure 8:**
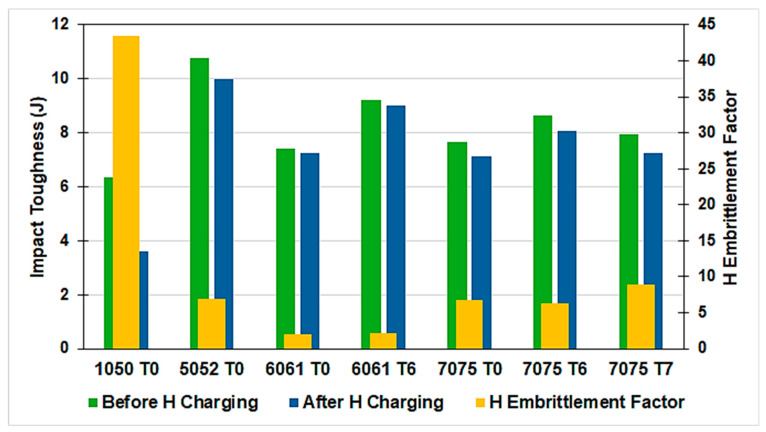
The behavior of hydrogen embrittlement of the Al alloys for hydrogen gas storage applications.

The results of the Charpy impact toughness test before and after H charging are presented in Figure 8. All alloys reveal a reduction in Charpy impact toughness value after H charging. This is attributed to hydrogen embrittlement. In the current work, the performance of the studied Al alloys for hydrogen gas storage applications was judged by estimating the hydrogen embrittlement factor using the Charpy impact toughness test. To evaluate the value of hydrogen embrittlement, the H embrittlement factor is taken as a percentage of the loss of Charpy impact toughness (U_l_) after H charging and calculated using the following equation:

U_l_ (%) = [(U_un_ − U_H_)/U_un_] × 100 (3)

where U_un_ is the Charpy impact toughness of the uncharged specimens and U_H_ is the Charpy impact toughness of the H-charged specimens.

As the value of the H embrittlement factor decreases, the alloy resistance to H embrittlement increases. As revealed in Figure 8, 6061 T0 and 6061 T6 Al alloys reveal the lowest H embrittlement factor. The highest H embrittlement factor occurs in 1050 T0 Al alloy, followed by 7075 T7 Al alloy. The 7075 T0, 7075 T6, and 5052 T0 Al alloys demonstrate a moderate and comparable H embrittlement factor.

**Figure 9:**
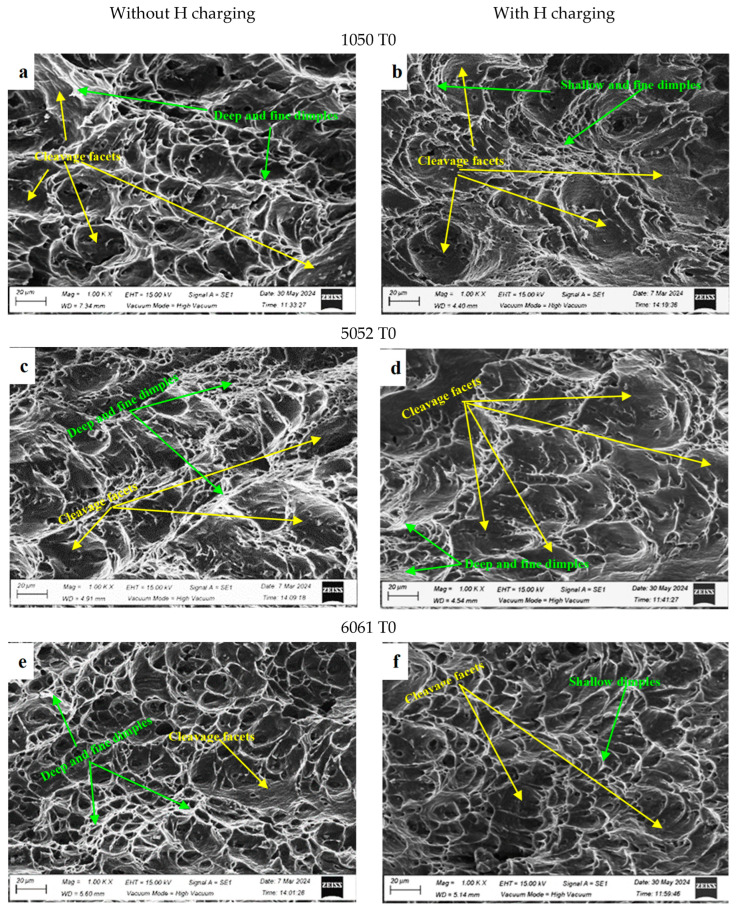

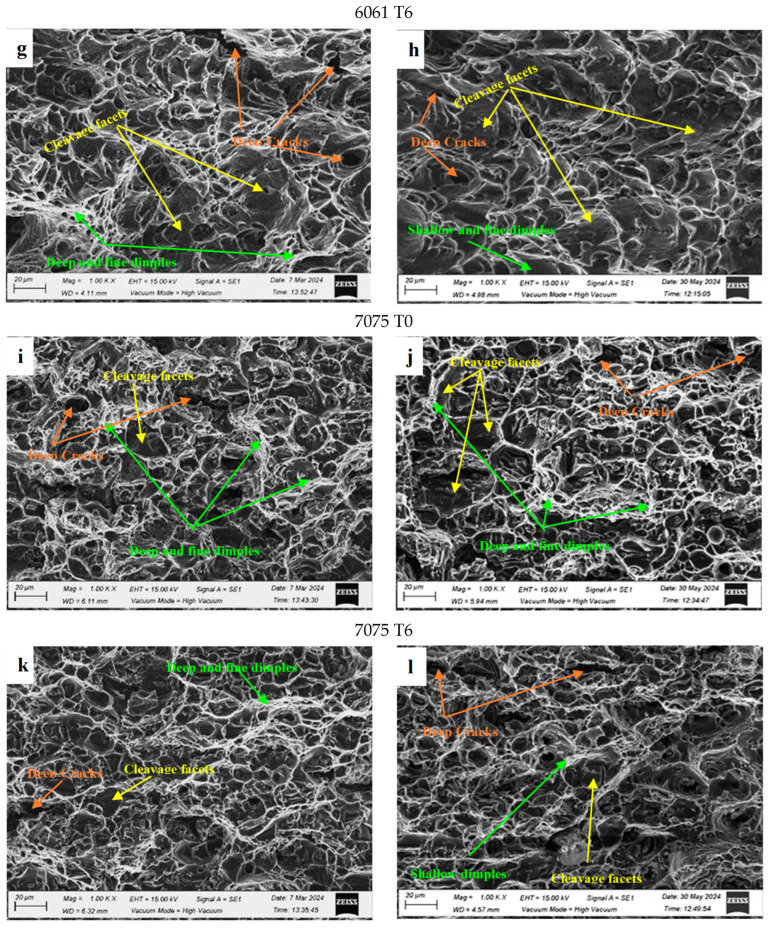

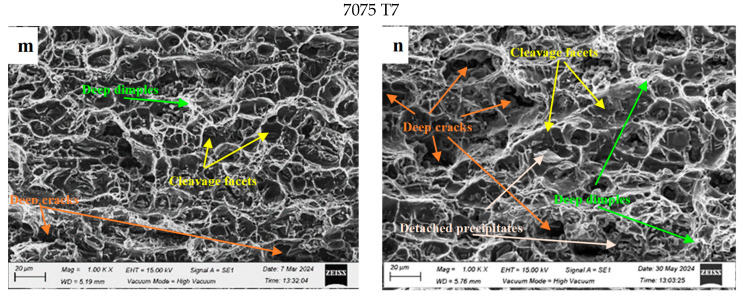
SEM images of the fracture surfaces after the Charpy impact test of the uncharged and H-charged Al alloys: (**a**,**c**,**e**,**g**,**i**,**k**,**m**) for the studied alloys without H charging and (**b**,**d**,**f**,**h**,**j**,**l**,**n**) for the studied alloys with H charging.

SEM analysis was performed on the fracture surfaces of the broken uncharged and H-charged Charpy impact specimens and presented in Figure 9. By investigating the fracture surfaces, it is possible to see how each alloy’s fracture attributes change depending on the alloy composition, temper conditions, and hydrogen charging. The structure–hydrogen interaction became more intense following H charging, which sped up the dislocation slip propagation and lowered the barriers to the dislocation emission [19]. All the fracture surfaces before and after H charging reveal mixed fracture mode mechanisms (Figure 9). The dimples, voids, and transgranular cracks are all attributes of the ductile fracture representing the hydrogen-enhanced localized plasticity (HELP) fracture mechanism, while the cleavage facet is the main feature of the brittle fracture representing the hydrogen-enhanced decohesion (HEDE) fracture mechanism [17]. It is obvious that the area fraction of the dimples decreases and the area fraction of cleavage facets increases in all fracture surfaces at different extents after H charging.

In 1050 T0, the fracture mode displays shallow dimples and more cleavage areas after hydrogen charging, as shown in Figure 9a,b. A similar observation was reported by Dey and Chattoraj for pure aluminum [20]. The 5052 T0 Al alloy also shows more cleavage areas after H charging (Figure 9c,d), but at a lower extent as compared to the 1050 T0 Al alloy. In 6061 T0 and 6061 T6 Al alloys, a mixed ductile-brittle fracture mode was observed before and after H charging, with more cleavage facets and shallow dimples after H charging as shown in Figure 9e,f, and Figure 9g,h, respectively. In 7075 Al alloys, the failure mode transfers from transgranular to intergranular (Figure 9i–n). Moreover, deep dimples are observed in many fracture surfaces, especially before H charging, indicating areas of localized plasticity. More coarse precipitates, voids, and deeper cracks appear in the 7075 T7 Al alloy after hydrogen charging (Figure 9n) as compared to the 7075 T0 and 7075 T6 Al alloys (Figure 9j,l). Therefore, multiple hydrogen embrittlement mechanisms are found to operate together under impact loading; this observation was reported previously by Bal et al. [17].

Zhao et al. [27] and Moshtaghi et al. [5] stated that grain boundaries can serve as hydrogen trap sites. They [5,27] emphasize the importance of hydrogen traps in reducing cracking. As hydrogen sources, the grain boundaries serve as relatively powerful trap sites that may gather hydrogen from the weaker ones. The density of hydrogen sources that can supply grain boundaries with hydrogen rises as a result of the segregation of the solutes close to grain boundaries. According to the hydrogen-enhanced decohesion (HEDE) process, higher hydrogen accumulation in grain boundaries lowers the cohesive interatomic strength, causing atoms to separate during loading and the production of intergranular cracks [5].

From the fractography images for 1050 T0 and 5052 T0 Al alloys, the fracture surface reveals large grain size structure in contrast to other alloys. This means small grain boundary areas in these alloys; therefore, hydrogen entraps inside the matrix, causing transgranular brittle failure through the hydrogen-enhanced decohesion (HEDE) process.

Moshtaghi et al. [5] also stated that alloying elements, particularly magnesium, can serve as hydrogen trap sites. Despite the grain boundary decohesion being favored by the co-segregation of alloying elements and hydrogen, the hydrogen embrittlement is prevented by the strong partitioning of hydrogen into the second-phase particles, which extracts hydrogen from the matrix [27]. Bal et al. also stated [17] that depending on the alloy composition, hydrogen altered the crack propagation behavior and that hydrogen enhanced the average dislocation mobility by reducing the cohesive energy. These findings are in agreement with the current results. The Al alloys containing Mg, i.e., 5052, 6061, and 7075 reveal good resistance to hydrogen embrittlement. According to Yamabe et al. [18], hydrogen trapping happens at the intermetallic particles in the subsurface layer of 6061-T6 Al alloy. The primary constituents of the intermetallic particles are Si, Cr, and Fe. This suggests that intermetallic particles close to the surface prevented hydrogen from entering the deep core of 6061-T6 Al alloy. Because of this, 6061-T6 Al alloy has a very low effective hydrogen diffusivity and may potentially have a strong resistance to hydrogen embrittlement.

Correlating the Al alloys performance as a hydrogen storage material with the material and processing costs, it was found that 6061 T0 Al alloy is the best choice, followed by 6061 T6 Al alloy, as they reveal the higher hydrogen embrittlement resistance at a reasonable cost. The 7075 T0 and 7075 T6 Al alloys have less preference to be used as hydrogen storage materials due to their higher cost and lower performance compared to 6061 Al alloys. The 7075 T7 Al alloy requires higher cost due to the long processing cycle and results in weaker resistance to hydrogen embrittlement as compared to other temper conditions of 7075 Al alloys. The 1050 T0 Al alloy should be excluded from being used as a hydrogen storage material due to its weak hydrogen embrittlement resistance.

## 4. Conclusions

The current work aims to examine the potential of utilizing Al alloys as electrodes for hydrogen gas production and storage applications. Analyses and comparisons were conducted regarding investigating different Al alloys and temper conditions, namely, 1050-T0, 5052-T0, 6061-T0, 6061-T6, 7075-T0, 7075-T6, and 7075-T7. In the first part, these alloys were employed as electrodes in water electrolyzers, and their influence was monitored on the generated hydrogen flow rate, the energy consumption, and the electrolyzer efficiency. The following conclusions are drawn:

- The 6061-T0 Al alloy reveals the best performance with the highest hydrogen flow rate, lower energy consumption, and higher process efficiency at a reasonable cost, as compared to other studied Al alloys.
- The 1050-T0, 5052-T0, 7075-T0, and 7075-T7 Al alloys came in second after the 6061-T0 Al alloy to be used as electrodes for hydrogen generation as they display moderate hydrogen flow rate, moderate energy consumption, and moderate process efficiency.
- The 6061-T6 and 7075-T6 Al alloys show the most inferior performance with low hydrogen flow rate, high energy consumption, and low process efficiency.
- Increasing the surface areas of the anode and cathode has a significant influence on the hydrogen flow rate and output current, followed by increasing the surface area of the cathode electrode only. While increasing the surface area of the anode electrode only has a weak influence on the hydrogen flow rate.
- According to OAT sensitivity analysis, rising water electrolysis temperature is the most influencing factor that significantly enhances the process efficiency of the hydrogen generation process, followed by increasing the electrode surface area. Changing the electrode material has the weakest influence on efficiency improvement.

In the second part of this work, the Al alloys were examined against hydrogen embrittlement for hydrogen gas storage applications. Charpy impact toughness tests were conducted for each alloy before and after H charging. Moreover, SEM was used to investigate the fracture characteristics. The following conclusions are drawn:

- The 6061-T0 and 6061-T6 Al alloys reveal the highest resistance to hydrogen embrittlement with mixed modes of ductile and brittle fracture; therefore, these alloys are the best candidates to be employed as liners in hydrogen storage tanks.
- The 7075 T0, 7075 T6, and 5052 T0 Al alloys have a moderate and comparable resistance to hydrogen embrittlement as compared to 6061 Al alloys.
- The 1050 T0 Al alloy shows inferior resistance to hydrogen embrittlement with a large area of cleavage facets, and 7075 T7 Al alloy shows weak resistance to hydrogen embrittlement with a deteriorated fracture surface containing deep cracks and detached precipitates. Therefore, 1050 T0 and 7075 T7 Al alloys should be excluded from being used as a liner for hydrogen storage tanks.

## Figures and Tables

**Table 1:** Chemical composition of the studied Al alloys (wt.%).

	Element	Mg	Cu	Zn	Si	Ti	Cr	Fe	Mn	Al
Alloy										
1050		0.05	0.04	0.05	0.22	0.03	0.03	0.04	0.04	Balance
5052		2.47	0.04	0.05	0.08	0.03	0.15	0.08	0.1	Balance
6061		1.08	0.28	0.04	0.8	0.06	0.26	0.25	0.05	Balance
7075		2.1	1.67	5.1	0.4	0.17	0.21	0.5	0.18	Balance

**Table 2:** Cost of the studied Al alloys in the hot-rolled conditions in March 2024.

Al Alloy	Cost
1050	2.43 USD/Kg
5052	2.71 USD/Kg
6061	3.05 USD/Kg
7075	3.59 USD/Kg

**Table 3:** Electrode surface areas.

	Anode Surface Area (mm^2^)	Cathode Surface Area (mm^2^)
Case 1	50 × 40	50 × 40
Case 2	90 × 50	90 × 50
Case 3	90 × 50	50 × 40
Case 4	50 × 40	90 × 50

**Table 4:** The electrolyzer efficiencies at different electrode surface areas at room temperature and constant voltage.

Alloy	Case	Efficiency (%)
1050 T0	Case 1	12.62
	Case 2	17.69
	Case 3	8.34
	Case 4	13.40
5052 T0	Case 1	14.96
	Case 2	18.69
	Case 3	12.50
	Case 4	17.25
6061 T0	Case 1	15.09
	Case 2	20.23
	Case 3	11.58
	Case 4	13.28
7075 T0	Case 1	16.55
	Case 2	20.83
	Case 3	11.88
	Case 4	15.27

## Data Availability

The original contributions presented in the study are included in the article, further inquiries can be directed to the corresponding author.
